# On the Occasional Pulsation of the Veins

**Published:** 1845-03

**Authors:** 


*On the Occasional Pulsation of the Veins.*—It is not that pulsation of the jugular veins—which depends upon a reflux of part of the blood from the right cavities of the heart, in consequence of the imperfect closure of the tricuspid valve—that the following remarks are applicable. "Our present object," says M. Martin Solon, in his communication to the Royal Academy, "is to draw attention to a pulsatory movement that we have occasionally observed in the veins in the back of the hand—a movement, that is dependent upon an isochronous continuation of the arterial pulse, and which, therefore, truly deserves to be considered as a pulse of the veins." The first instance, in which he observed the phenomenon, was one of severe Pneumonia, after the patient had been largely bled. On the fifth day the dorsal veins of each hand were perceived alternately to swell up and to subside, as is the case with any superficial artery. The pulsations were distinctly isochronous with those at the wrist. It was abundantly obvious that the movements of the veins were quite independent of those of the neighbouring arteries, and of the agitation of the subjacent tendons. When the brachial artery was firmly compressed, the pulse in the veins, as well as in the radial and ulnar arteries, was at once suspended. In this instance, the phenomenon in question was observed for six or seven days successively. M. Solon expressed his conviction, that the venous pulse was in short nothing else but the continuation of the ordinary arterial one, communicated from the arteries directly to the veins. It is not improbable, he very justly remarks, that in order that this phenomenon may occur, the circulating fluids should be in a more attenuated condition than they are in a state of perfect health. That such is probable the case, is rendered still more likely by the results of the second case—one of typhoid fever—related by him.

Dr. Graves also has had occasion to observe a distinct pulsation of the veins in two cases of active inflammation, in both of which blood-letting had been already freely employed; and Dr. Ward, too, has seen it in a woman recently delivered. It would seem therefore that the phenomenon in question is decidedly connected with an attenuated state of the blood.

M. Cruveilhier stated that he had never seen any appearance of pulse in the veins of the hand, atthough the phenomenon in question was not at all uncommon in the veins at the bend of the arm. He had indeed more than once, in consequence of the vein in blood letting throwing out its contents in jets, been apprehensive that the artery was wounded: but such an accident had never occurred in his practice. He attributed the phenomenon of the venous pulse, in all cases, to subjacent arteries communicating their movement to the veins; and never to any direct transmission of their pulsations along their capillary extremities.

M. Velpeau alluded to a case of typhus fever, in which all the superficial veins in both upper extremities were moved with most distinct pulsations, that were certainly not owing to any shock communicated to them directly by subjacent or neighbouring arteries. On the whole, he was inclined to attribute the phenomenon to the reflux of the blood backwards from the heart, along the subclavian and brachial veins. But, as M. Solon remarked, how could this explanation hold in his two cases, where there was no pulsation of the brachial and anti-brachial veins, but only of those of the hands?

M. Blandin expressed his entire concurrence in the opinions of M. Solon. He alluded to the circumstance that, two centuries ago, Harvey himself had distinctly asserted that the heart will sometimes transmit its pulsations even to the veins: and he then mentioned some experiments which M. Magendie and he had performed, and which tended to establish the truth of this positiion. If a part of the circulatory system be emptied, (in the dead body) by means of a first injection, pushed in with force, and then a second injection be thrown in, by jerks, into a large arterial trunk, we shall find that it will escape from a wound in any one of the principal veins of that system, in jets, as from an artery. If this can be done in the dead body, why should we deny the possible occurrence of it in the living?

M. Dubois intimated his dissent from these views, and agreed with M. Cruveilhier in ascribing all venous pulsations, either to the shocks communicated from the contiguous arteries, or (in some cases) to the reflux of the blood along the jugular.—*Ibid, from Comptes Rendus.*

